# Supplementary material for: Transmembrane Domain Dominance Drives Emergent Signaling and Allosteric Inversion in mGlu1/5 Heterodimers
Source: bioRxiv. 2026 May 22:2026.05.20.726619. Preprint. [Version 1] doi: 10.64898/2026.05.20.726619 (PMC13228339; doi:10.64898/2026.05.20.726619)
Supplement: Supplement 1 [file media-1.pdf]

Supplemental Information

Construct	Primary Structure
ALFA-rmGlu$_1$ WT	MVLLLILSVLLLKEDVRGSAQSTRPSRLEEELRRRLTEPDKLASSQRSVARMDGDVIIGALFSVHHQPPAEKVPERKCGEIREQYGIQRVEAMFHTLDKINADPVLLPNITLGSEIRD<br>SCWHSSVALEQSIEFIRDSLISIRDEKDGLNRCLPDGQTLPPGRTKKPIAGVIGPGSSSVAIQVQNLLQLFDIPQIAYSATSIDLSDKTLYKYFLRVVPSDTLQARAMLDIVKRYNWTY<br>VSAVHTEGNYGESGMDAFKELAAQEGLCIAHSDKIYSNAGEKSFDRLLRKLRERLPKARVVVCFCEGMTVRGLLSAMRRLGVVGEFSLIGSDGWADRDEVIEGYEVEANGGITIK<br>LQSPEVRSFDDYFLKLRLDTNTRNPWFPEFWQHRFQCRLPGHLLENPNFKKVCTGNESLEENYVQDSKMGFVINAIYAMAHGLQNMHHALCPGHVGLCDAMKPIDGRKLLDFLI<br>KSSFVGVSGEEVWFDEKGDAPGRYDIMNLQYTEANRYDYVHVGTWHEGVLNIDDYKIQMNKSGMVRSVCSEPCLKGQIKVIRKGEVSCCWICTACKENEFVQDEFTCRACDLG<br>WWPNAELTGCEPIPVRYLEWSDIESIIAIAFSCLGILVTLFVTLIFVLYRDTPVVKSSSRELCYIILAGIFLGYVCPFTLIAKPTTTSCYLQRLLVGLSSAMCYSALVTKTNRIARILAGSKK<br>KICTRKPRFMSAWAQVIIASILISVQLTLVVTLIIMEPPMPILSYPSIKEVYLICNTSNLGVVAPVGYNGLLIMSCTYYAFKTRNVPANFNEAKYIAFTMYTTCIIWLAFVPIYFGSNYKIITT<br>CFAVSLSVTVALGCMFTPKMYIIIAKPERNVRSAFTTSDVVRMHVGDGKLPCRSNTFLNIFRRKKPGAGNANSNGKSVSWSEPGGRQAPKGQHVWQRLSVHVKTNETACNQTAV<br>IKPLTKSYQGSGKSLTFSDASTKTLYNVEEEDNTPSAHFSPPSSPSMVVHRRGPPVATTPPLPPHLTAEETPLFLADSVIPKGLPPPLPQQQPQQPPPQQPPQQPKSLMDQLQGV<br>VTNFGSGIPDFHAVLAGPGTPGNSLRSLYPPPPPPQHLQMLPLHLSTFQEESISPPGEDIDDDSERFKLLQEFVYEREGNTEEDELEEEEDLPTASKLTPEDSPALTPPSPFRDSV<br>ASGSSVPSSPVSESVLCTPPNVTYASVILRDYKQSSSTL
ALFA-rmGlu$_1$ -HiBiT	MVLLLILSVLLLKEDVRGSAQSTRPSRLEEELRRRLTEPDKLASSQRSVARMDGDVIIGALFSVHHQPPAEKVPERKCGEIREQYGIQRVEAMFHTLDKINADPVLLPNITLGSEIRD<br>SCWHSSVALEQSIEFIRDSLISIRDEKDGLNRCLPDGQTLPPGRTKKPIAGVIGPGSSSVAIQVQNLLQLFDIPQIAYSATSIDLSDKTLYKYFLRVVPSDTLQARAMLDIVKRYNWTY<br>VSAVHTEGNYGESGMDAFKELAAQEGLCIAHSDKIYSNAGEKSFDRLLRKLRERLPKARVVVCFCEGMTVRGLLSAMRRLGVVGEFSLIGSDGWADRDEVIEGYEVEANGGITIK<br>LQSPEVRSFDDYFLKLRLDTNTRNPWFPEFWQHRFQCRLPGHLLENPNFKKVCTGNESLEENYVQDSKMGFVINAIYAMAHGLQNMHHALCPGHVGLCDAMKPIDGRKLLDFLI<br>KSSFVGVSGEEVWFDEKGDAPGRYDIMNLQYTEANRYDYVHVGTWHEGVLNIDDYKIQMNKSGMVRSVCSEPCLKGQIKVIRKGEVSCCWICTACKENEFVQDEFTCRACDLG<br>WWPNAELTGCEPIPVRYLEWSDIESIIAIAFSCLGILVTLFVTLIFVLYRDTPVVKSSSRELCYIILAGIFLGYVCPFTLIAKPTTTSCYLQRLLVGLSSAMCYSALVTKTNRIARILAGSKK<br>KICTRKPRFMSAWAQVIIASILISVQLTLVVTLIIMEPPMPILSYPSIKEVYLICNTSNLGVVAPVGYNGLLIMSCTYYAFKTRNVPANFNEAKYIAFTMYTTCIIWLAFVPIYFGSNYKIITT<br>CFAVSLSVTVALGCMFTPKMYIIIAKPERNVRSAFTTSDVVRMHVGDGKLPCRSNTFLNIFRRKKPGAGNAGGGGSVSGWRLFKKISSGGGGNSNGKSVSWSEPGGRQAPKGQ<br>HVWQRLSVHVKTNETACNQTAVIKPLTKSYQGSGKSLTFSDASTKTLYNVEEEDNTPSAHFSPPSSPSMVVHRRGPPVATTPPLPPHLTAEETPLFLADSVIPKGLPPPLPQQQP<br>QQPPPQQPPQQPKSLMDQLQGVVTNFGSGIPDFHAVLAGPGTPGNSLRSLYPPPPPPQHLQMLPLHLSTFQEESISPPGEDIDDDSERFKLLQEFVYEREGNTEEDELEEEEDLP<br>TASKLTPEDSPALTPPSPFRDSVASGSSVPSSPVSESVLCTPPNVTYASVILRDYKQSSSTL
ALFA-rmGlu$_1$ -LgBiT	MVLLLILSVLLLKEDVRGSAQSTRPSRLEEELRRRLTEPDKLASSQRSVARMDGDVIIGALFSVHHQPPAEKVPERKCGEIREQYGIQRVEAMFHTLDKINADPVLLPNITLGSEIRD<br>SCWHSSVALEQSIEFIRDSLISIRDEKDGLNRCLPDGQTLPPGRTKKPIAGVIGPGSSSVAIQVQNLLQLFDIPQIAYSATSIDLSDKTLYKYFLRVVPSDTLQARAMLDIVKRYNWTY<br>VSAVHTEGNYGESGMDAFKELAAQEGLCIAHSDKIYSNAGEKSFDRLLRKLRERLPKARVVVCFCEGMTVRGLLSAMRRLGVVGEFSLIGSDGWADRDEVIEGYEVEANGGITIK<br>LQSPEVRSFDDYFLKLRLDTNTRNPWFPEFWQHRFQCRLPGHLLENPNFKKVCTGNESLEENYVQDSKMGFVINAIYAMAHGLQNMHHALCPGHVGLCDAMKPIDGRKLLDFLI<br>KSSFVGVSGEEVWFDEKGDAPGRYDIMNLQYTEANRYDYVHVGTWHEGVLNIDDYKIQMNKSGMVRSVCSEPCLKGQIKVIRKGEVSCCWICTACKENEFVQDEFTCRACDLG<br>WWPNAELTGCEPIPVRYLEWSDIESIIAIAFSCLGILVTLFVTLIFVLYRDTPVVKSSSRELCYIILAGIFLGYVCPFTLIAKPTTTSCYLQRLLVGLSSAMCYSALVTKTNRIARILAGSKK<br>KICTRKPRFMSAWAQVIIASILISVQLTLVVTLIIMEPPMPILSYPSIKEVYLICNTSNLGVVAPVGYNGLLIMSCTYYAFKTRNVPANFNEAKYIAFTMYTTCIIWLAFVPIYFGSNYKIITT<br>CFAVSLSVTVALGCMFTPKMYIIIAKPERNVRSAFTTSDVVRMHVGDGKLPCRSNTFLNIFRRKKPGAGNANSNGKSVSWSEPGGRQAPKGQHVWQRLSVHVKTNETACNQTAV<br>IKPLTKSYQGSGKSLTFSDASTKTLYNVEEEDNTPSAHFSPPSSPSMVVHRRGPPVATTPPLPPHLTAEETPLFLADSVIPKGLPPPLPQQQPQQPPPQQPPQQPKSLMDQLQGV<br>VTNFGSGIPDFHAVLAGPGTPGNSLRSLYPPPPPPQHLQMLPLHLSTFQEESISPPGEDIDDDSERFKLLQEFVYEREGNTEEDELEEEEDLPTASKLTPEDSPALTPPSPFRDSV<br>ASGSSVPSSPVSESVLCTPPNVTYASVILRDYKQSSSTLGSPPARATLEVFTLEDFVGDWEQTAAYNLDQVLEQGGVSSLLQNLAVSVTPIQRIVRSGENALKIDIHVIIPYEGLSAD<br>QMAQIEEVFKVVYPVDDHHFKVILPYGTLVIDGVTPNMLNYFGRPYEGIAVFDGKKITVTGTLWNGNKIIDERLITPDGSMLFRVTINS
ALFA-rmGlu$_5$ WT	MVLLLILSVLLLKEDVRGSAQSTRPSRLEEELRRRLTEPDKLQSSERRVVAHMPGDIIIGALFSVHHQPTVDKVHERKCGAVREQYGIQRVEAMLHTLERINSDPTLLPNITLGCEIR<br>DSCWHSAVALEQSIEFIRDSLISSEEEEGLVRCVDGSSSFRSKKPIVGVIGPGSSSVAIQVQNLLQLFNIPQIAYSATSMDLSDKTLFKYFMRVVPSDAQQARAMVDIVKRYNWTYV<br>SAVHTEGNYGESGMEAFKDMSAKEGICIAHSYKIYSNAGEQSFDKLLKKLRSHLPKARVVACFCEGMTVRGLLMAMRRLGLAGEFLLLGSDGWADRYDVTDGYQREAVGGITIKL<br>QSPDVKWFDDYYLKLRPETNLRNPWFQEFWQHRFQCRLEGFAQENSKYNKTCNSSLTLRTHHVQDSKMGFVINAIYSMAYGLHNMQMSLCPGYAGLCDAMKPIDGRKLLDSL<br>MKTNFTGVSGDMILFDENGDSPGRYEIMNFKEMGKDYFDYINVGSWDNGELKMDDDEVWSKKNNIIRSVCSEPCEKGQIKVIRKGEVSCCWTCTPCKENEYVFDEYTCKACQLG<br>SWPTDDLTGCDLIPVQYLRWGDPEPIAAVVFACLGLLATLFVTVIFIIYRDTPVVKSSSRELCYIILAGICLGYLCTFCLIAKPKQIYCYLQRIGIGLSPAMSYSALVTKTNRIARILAGSKK<br>KICTKKPRFMSACAQLVIAFILICIQLGIIVALFIMEPPDIMHDYPSIREVYLICNTTNLGVVTPLGYNGLLILSCTFYAFKTRNVPANFNEAKYIAFTMYTTCIIWLAFVPIYFGSNYKIITMC<br>FSVSLSATVALGCMFVPKVYIILAKPERNVRSAFTTSTVVRMHVGDGKSSSAASRSSSLVNLWKRRGSSGETLSSNGKSVTWAQNEKSTRGQHLWQRLSVHINKKENPNQTAVIK<br>PFPKSTENRGPGAAAGGGSGPGVAGAGNAGCTATGGPEPPDAGPKALYDVAEAEESFPAAARPRSPSPISTLSHLAGSAGRTDDDAPSLHSETAARSSSSQGSLMEQISSVVT<br>RFTANISELNSMMLSTAATPGPPGTPICSSYLIPKEIQLPTTMTTFAEIQPLPAIEVTGGAQGATGVSPAQETPTGAESAPGKPDLEELVALTPPSPFRDSVDSGSTTPNSPVSESAL<br>CIPSSPKYDTLIIRDYTQSSSSL
ALFA-rmGlu$_5$ -HiBiT	MVLLLILSVLLLKEDVRGSAQSTRPSRLEEELRRRLTEPDKLQSSERRVVAHMPGDIIIGALFSVHHQPTVDKVHERKCGAVREQYGIQRVEAMLHTLERINSDPTLLPNITLGCEIR<br>DSCWHSAVALEQSIEFIRDSLISSEEEEGLVRCVDGSSSFRSKKPIVGVIGPGSSSVAIQVQNLLQLFNIPQIAYSATSMDLSDKTLFKYFMRVVPSDAQQARAMVDIVKRYNWTYV<br>SAVHTEGNYGESGMEAFKDMSAKEGICIAHSYKIYSNAGEQSFDKLLKKLRSHLPKARVVACFCEGMTVRGLLMAMRRLGLAGEFLLLGSDGWADRYDVTDGYQREAVGGITIKL<br>QSPDVKWFDDYYLKLRPETNLRNPWFQEFWQHRFQCRLEGFAQENSKYNKTCNSSLTLRTHHVQDSKMGFVINAIYSMAYGLHNMQMSLCPGYAGLCDAMKPIDGRKLLDSL<br>MKTNFTGVSGDMILFDENGDSPGRYEIMNFKEMGKDYFDYINVGSWDNGELKMDDDEVWSKKNNIIRSVCSEPCEKGQIKVIRKGEVSCCWTCTPCKENEYVFDEYTCKACQLG<br>SWPTDDLTGCDLIPVQYLRWGDPEPIAAVVFACLGLLATLFVTVIFIIYRDTPVVKSSSRELCYIILAGICLGYLCTFCLIAKPKQIYCYLQRIGIGLSPAMSYSALVTKTNRIARILAGSKK<br>KICTKKPRFMSACAQLVIAFILICIQLGIIVALFIMEPPDIMHDYPSIREVYLICNTTNLGVVTPLGYNGLLILSCTFYAFKTRNVPANFNEAKYIAFTMYTTCIIWLAFVPIYFGSNYKIITMC<br>FSVSLSATVALGCMFVPKVYIILAKPERNVRSAFTTSTVVRMHVGDGKSSSAASRSSSLVNLWKRRGSSGGGGGSVSGWRLFKKISSGGGGETLSSNGKSVTWAQNEKSTRGQ<br>HLWQRLSVHINKKENPNQTAVIKPFPKSTENRGPGAAAGGGSGPGVAGAGNAGCTATGGPEPPDAGPKALYDVAEAEESFPAAARPRSPSPISTLSHLAGSAGRTDDDAPSLHS<br>ETAARSSSSQGSLMEQISSVVTRFTANISELNSMMLSTAATPGPPGTPICSSYLIPKEIQLPTTMTTFAEIQPLPAIEVTGGAQGATGVSPAQETPTGAESAPGKPDLEELVALTPPS<br>PFRDSVDSGSTTPNSPVSESALCIPSSPKYDTLIIRDYTQSSSSL
ALFA-rmGlu$_5$ -LgBiT	MVLLLILSVLLLKEDVRGSAQSTRPSRLEEELRRRLTEPDKLQSSERRVVAHMPGDIIIGALFSVHHQPTVDKVHERKCGAVREQYGIQRVEAMLHTLERINSDPTLLPNITLGCEIR<br>DSCWHSAVALEQSIEFIRDSLISSEEEEGLVRCVDGSSSFRSKKPIVGVIGPGSSSVAIQVQNLLQLFNIPQIAYSATSMDLSDKTLFKYFMRVVPSDAQQARAMVDIVKRYNWTYV<br>SAVHTEGNYGESGMEAFKDMSAKEGICIAHSYKIYSNAGEQSFDKLLKKLRSHLPKARVVACFCEGMTVRGLLMAMRRLGLAGEFLLLGSDGWADRYDVTDGYQREAVGGITIKL<br>QSPDVKWFDDYYLKLRPETNLRNPWFQEFWQHRFQCRLEGFAQENSKYNKTCNSSLTLRTHHVQDSKMGFVINAIYSMAYGLHNMQMSLCPGYAGLCDAMKPIDGRKLLDSL<br>MKTNFTGVSGDMILFDENGDSPGRYEIMNFKEMGKDYFDYINVGSWDNGELKMDDDEVWSKKNNIIRSVCSEPCEKGQIKVIRKGEVSCCWTCTPCKENEYVFDEYTCKACQLG<br>SWPTDDLTGCDLIPVQYLRWGDPEPIAAVVFACLGLLATLFVTVIFIIYRDTPVVKSSSRELCYIILAGICLGYLCTFCLIAKPKQIYCYLQRIGIGLSPAMSYSALVTKTNRIARILAGSKK<br>KICTKKPRFMSACAQLVIAFILICIQLGIIVALFIMEPPDIMHDYPSIREVYLICNTTNLGVVTPLGYNGLLILSCTFYAFKTRNVPANFNEAKYIAFTMYTTCIIWLAFVPIYFGSNYKIITMC<br>FSVSLSATVALGCMFVPKVYIILAKPERNVRSAFTTSTVVRMHVGDGKSSSAASRSSSLVNLWKRRGSSGETLSSNGKSVTWAQNEKSTRGQHLWQRLSVHINKKENPNQTAVIK<br>PFPKSTENRGPGAAAGGGSGPGVAGAGNAGCTATGGPEPPDAGPKALYDVAEAEESFPAAARPRSPSPISTLSHLAGSAGRTDDDAPSLHSETAARSSSSQGSLMEQISSVVT<br>RFTANISELNSMMLSTAATPGPPGTPICSSYLIPKEIQLPTTMTTFAEIQPLPAIEVTGGAQGATGVSPAQETPTGAESAPGKPDLEELVALTPPSPFRDSVDSGSTTPNSPVSESAL<br>CIPSSPKYDTLIIRDYTQSSSSLGSPPARATLEVFTLEDFVGDWEQTAAYNLDQVLEQGGVSSLLQNLAVSVTPIQRIVRSGENALKIDIHVIIPYEGLSADQMAQIEEVFKVVYPVDD<br>HHFKVILPYGTLVIDGVTPNMLNYFGRPYEGIAVFDGKKITVTGTLWNGNKIIDERLITPDGSMLFRVTINS
Chimeras ALFA-rmGlu$_5^{1ECD}$ -HiBiT or ALFA-rmGlu$_1^{5TM}$ -HiBiT	MVLLLILSVLLLKEDVRGSAQSTRPSRLEEELRRRLTEPDKLASSQRSVARMDGDVIIGALFSVHHQPPAEKVPERKCGEIREQYGIQRVEAMFHTLDKINADPVLLPNITLGSEIRD<br>SCWHSSVALEQSIEFIRDSLISIRDEKDGLNRCLPDGQTLPPGRTKKPIAGVIGPGSSSVAIQVQNLLQLFDIPQIAYSATSIDLSDKTLYKYFLRVVPSDTLQARAMLDIVKRYNWTY<br>VSAVHTEGNYGESGMDAFKELAAQEGLCIAHSDKIYSNAGEKSFDRLLRKLRERLPKARVVVCFCEGMTVRGLLSAMRRLGVVGEFSLIGSDGWADRDEVIEGYEVEANGGITIK<br>LQSPEVRSFDDYFLKLRLDTNTRNPWFPEFWQHRFQCRLPGHLLENPNFKKVCTGNESLEENYVQDSKMGFVINAIYAMAHGLQNMHHALCPGHVGLCDAMKPIDGRKLLDFLI<br>KSSFVGVSGEEVWFDEKGDAPGRYDIMNLQYTEANRYDYVHVGTWHEGVLNIDDYKIQMNKSGMVRSVCSEPCEKGQIKVIRKGEVSCCWTCTPCKENEYVFDEYTCKACQLG<br>SWPTDDLTGCDLIPVQYLRWGDPEPIAAVVFACLGLLATLFVTVIFIIYRDTPVVKSSSRELCYIILAGICLGYLCTFCLIAKPKQIYCYLQRIGIGLSPAMSYSALVTKTNRIARILAGSKK<br>KICTKKPRFMSACAQLVIAFILICIQLGIIVALFIMEPPDIMHDYPSIREVYLICNTTNLGVVTPLGYNGLLILSCTFYAFKTRNVPANFNEAKYIAFTMYTTCIIWLAFVPIYFGSNYKIITMC<br>FSVSLSATVALGCMFVPKVYIILAKPERNVRSAFTTSTVVRMHVGDGKSSSAASRSSSLVNLWKRRGSSGGGGGSVSGWRLFKKISSGGGGETLSSNGKSVTWAQNEKSTRGQ<br>HLWQRLSVHINKKENPNQTAVIKPFPKSTENRGPGAAAGGGSGPGVAGAGNAGCTATGGPEPPDAGPKALYDVAEAEESFPAAARPRSPSPISTLSHLAGSAGRTDDDAPSLHS<br>ETAARSSSSQGSLMEQISSVVTRFTANISELNSMMLSTAATPGPPGTPICSSYLIPKEIQLPTTMTTFAEIQPLPAIEVTGGAQGATGVSPAQETPTGAESAPGKPDLEELVALTPPS<br>PFRDSVDSGSTTPNSPVSESALCIPSSPKYDTLIIRDYTQSSSSL

Construct	Primary Structure
Chimeras ALFA-rmGlu$_1^{5ECD}$ -LgBiT or ALFA-rmGlu$_5^{1TM}$ -LgBiT	MVLLLILSVLLLKEDVRGSAQSTRPSRLEEELRRRLTEPDKLQSSERRVVAHMPGDIIIGALFSVHHQPTVDKVHERKCGAVREQYGIQRVEAMLHTLERINSDPTLLPNITLGCEIR<br>DSCWHSAVALEQSIEFIRDSLISSEEEEGLVRCVDGSSSFRSKKPIVGVIGPGSSSVAIQVQNLLQLFNIPQIAYSATSMDLSDKTLFKYFMRVVPSDAQQARAMVDIVKRYNWTYV<br>SAVHTEGNYGESGMEAFKDMSAKEGICIAHSYKIYSNAGEQSFDKLLKKLRSHLPKARVVACFCEGMTVRGLLMAMRRLGLAGEFLLLGSDGWADRYDVTDGYQREAVGGITIKL<br>QSPDVKWFDDYYLKLRPETNLRNPWFQEFWQHRFQCRLEGFAQENSKYNKTCNSSLTLRTHHVQDSKMGFVINAIYSMAYGLHNMQMSLCPGYAGLCDAMKPIDGRKLLDSL<br>MKTNFTGVSGDMILFDENGDSPGRYEIMNFKEMGKDYFDYINVGSWDNGELKMDDDEVWSKKNNIIRSVCSEPCLKGQIKVIRKGEVSCCWICTACKENEFVQDEFTCRACDLG<br>WWPNAELTGCEPIPVRYLEWSDIESIIAIAFSCLGILVTLFVTLIFVLYRDTPVVKSSSRELCYIILAGIFLGYVCPFTLIAKPTTTSCYLQRLLVGLSSAMCYSALVTKTNRIARILAGSKK<br>KICTRKPRFMSAWAQVIIASILISVQLTLVVTLIIMEPPMPILSYPSIKEVYLICNTSNLGVVAPVGYNGLLIMSCTYYAFKTRNVPANFNEAKYIAFTMYTTCIIWLAFVPIYFGSNYKIITT<br>CFAVSLSVTVALGCMFTPKMYIIIAKPERNVRSAFTTSDVVRMHVGDGKLPCRSNTFLNIFRRKKPGAGNANSNGKSVSWSEPGGRQAPKGQHVWQRLSVHVKTNETACNQTAV<br>IKPLTKSYQGSGKSLTFSDASTKTLYNVEEEDNTPSAHFSPPSSPSMVVHRRGPPVATTPPLPPHLTAEETPLFLADSVIPKGLPPPLPQQQPQQPPPQQPPQQPKSLMDQLQGV<br>VTNFGSGIPDFHAVLAGPGTPGNSLRSLYPPPPPPQHLQMLPLHLSTFQEESISPPGEDIDDDSERFKLLQEFVYEREGNTEEDELEEEEDLPTASKLTPEDSPALTPPSPFRDSV<br>ASGSSVPSSPVSESVLCTPPNVTYASVILRDYKQSSSTLGSPPARATLEVFTLEDFVGDWEQTAAYNLDQVLEQGGVSSLLQNLAVSVTPIQRIVRSGENALKIDIHVIIPYEGLSAD<br>QMAQIEEVFKVVYPVDDHHFKVILPYGTLVIDGVTPNMLNYFGRPYEGIAVFDGKKITVTGTLWNGNKIIDERLITPDGSMLFRVTINS
HA-rmGlu$_1$-GB1	MVRLLLIFFPMIFLEMSILPRYPYDVPDYAMPDRKVLLAGASSQRSVARMDGDVIIGALFSVHHQPPAEKVPERKCGEIREQYGIQRVEAMFHTLDKINADPVLLPNITLGSEIRDSC<br>WHSSVALEQSIEFIRDSLISIRDEKDGLNRCLPDGQTLPPGRTKKPIAGVIGPGSSSVAIQVQNLLQLFDIPQIAYSATSIDLSDKTLYKYFLRVVPSDTLQARAMLDIVKRYNWTYVSA<br>VHTEGNYGESGMDAFKELAAQEGLCIAHSDKIYSNAGEKSFDRLLRKLRERLPKARVVVCFCEGMTVRGLLSAMRRLGVVGEFSLIGSDGWADRDEVIEGYEVEANGGITIKLQS<br>PEVRSFDDYFLKLRLDTNTRNPWFPEFWQHRFQCRLPGHLLENPNFKKVCTGNESLEENYVQDSKMGFVINAIYAMAHGLQNMHHALCPGHVGLCDAMKPIDGRKLLDFLIKSS<br>FVGVSGEEVWFDEKGDAPGRYDIMNLQYTEANRYDYVHVGTWHEGVLNIDDYKIQMNKSGMVRSVCSEPCLKGQIKVIRKGEVSCCWICTACKENEFVQDEFTCRACDLGWWP<br>NAELTGCEPIPVRYLEWSDIESIIAIAFSCLGILVTLFVTLIFVLYRDTPVVKSSSRELCYIILAGIFLGYVCPFTLIAKPTTTSCYLQRLLVGLSSAMCYSALVTKTNRIARILAGSKKKICT<br>RKPRFMSAWAQVIIASILISVQLTLVVTLIIMEPPMPILSYPSIKEVYLICNTSNLGVVAPVGYNGLLIMSCTYYAFKTRNVPANFNEAKYIAFTMYTTCIIWLAFVPIYFGSNYKIITTCFAV<br>SLSVTVALGCMFTPKMYIIIAKPERNVRSAFTTSDVVRMHAAATGSSTNNNEEEKSRLLEKENRELEKIIAEKEERVSELRHQLQSRQQLKKTN*
HA-rmGlu$_1$-GB2	MVRLLLIFFPMIFLEMSILPRYPYDVPDYAMPDRKVLLAGASSQRSVARMDGDVIIGALFSVHHQPPAEKVPERKCGEIREQYGIQRVEAMFHTLDKINADPVLLPNITLGSEIRDSC<br>WHSSVALEQSIEFIRDSLISIRDEKDGLNRCLPDGQTLPPGRTKKPIAGVIGPGSSSVAIQVQNLLQLFDIPQIAYSATSIDLSDKTLYKYFLRVVPSDTLQARAMLDIVKRYNWTYVSA<br>VHTEGNYGESGMDAFKELAAQEGLCIAHSDKIYSNAGEKSFDRLLRKLRERLPKARVVVCFCEGMTVRGLLSAMRRLGVVGEFSLIGSDGWADRDEVIEGYEVEANGGITIKLQS<br>PEVRSFDDYFLKLRLDTNTRNPWFPEFWQHRFQCRLPGHLLENPNFKKVCTGNESLEENYVQDSKMGFVINAIYAMAHGLQNMHHALCPGHVGLCDAMKPIDGRKLLDFLIKSS<br>FVGVSGEEVWFDEKGDAPGRYDIMNLQYTEANRYDYVHVGTWHEGVLNIDDYKIQMNKSGMVRSVCSEPCLKGQIKVIRKGEVSCCWICTACKENEFVQDEFTCRACDLGWWP<br>NAELTGCEPIPVRYLEWSDIESIIAIAFSCLGILVTLFVTLIFVLYRDTPVVKSSSRELCYIILAGIFLGYVCPFTLIAKPTTTSCYLQRLLVGLSSAMCYSALVTKTNRIARILAGSKKKICT<br>RKPRFMSAWAQVIIASILISVQLTLVVTLIIMEPPMPILSYPSIKEVYLICNTSNLGVVAPVGYNGLLIMSCTYYAFKTRNVPANFNEAKYIAFTMYTTCIIWLAFVPIYFGSNYKIITTCFAV<br>SLSVTVALGCMFTPKMYIIIAKPERNVRSAFTTSDVVRMHAAATSTSVTSVNQASTSRLEGLQSENHRLRMKITELDKDLEEVTMQLQDTPEKKTN*
FLAG-rmGlu$_5$-GB1	MVLLLILSVLLLKEDVRGSAQSDYKDDDDKSERRVVAHMPGDIIIGALFSVHHQPTVDKVHERKCGAVREQYGIQRVEAMLHTLERINSDPTLLPNITLGCEIRDSCWHSAVALEQSI<br>EFIRDSLISSEEEEGLVRCVDGSSSFRSKKPIVGVIGPGSSSVAIQVQNLLQLFNIPQIAYSATSMDLSDKTLFKYFMRVVPSDAQQARAMVDIVKRYNWTYVSAVHTEGNYGESGM<br>EAFKDMSAKEGICIAHSYKIYSNAGEQSFDKLLKKLRSHLPKARVVACFCEGMTVRGLLMAMRRLGLAGEFLLLGSDGWADRYDVTDGYQREAVGGITIKLQSPDVKWFDDYYLK<br>LRPETNLRNPWFQEFWQHRFQCRLEGFAQENSKYNKTCNSSLTLRTHHVQDSKMGFVINAIYSMAYGLHNMQMSLCPGYAGLCDAMKPIDGRKLLDSLMKTNFTGVSGDMILF<br>DENGDSPGRYEIMNFKEMGKDYFDYINVGSWDNGELKMDDDEVWSKKNNIIRSVCSEPCEKGQIKVIRKGEVSCCWTCTPCKENEYVFDEYTCKACQLGSWPTDDLTGCDLIPV<br>QYLRWGDPEPIAAVVFACLGLLATLFVTVIFIIYRDTPVVKSSSRELCYIILAGICLGYLCTFCLIAKPKQIYCYLQRIGIGLSPAMSYSALVTKTNRIARILAGSKKKICTKKPRFMSACAQ<br>LVIAFILICIQLGIIVALFIMEPPDIMHDYPSIREVYLICNTTNLGVVTPLGYNGLLILSCTFYAFKTRNVPANFNEAKYIAFTMYTTCIIWLAFVPIYFGSNYKIITMCFSVSLSATVALGCMF<br>VPKVYIILAKPERNVRSAFTTSTVVRMHAAATGSSTNNNEEEKSRLLEKENRELEKIIAEKEERVSELRHQLQSRQQLKKTN*
FLAG-rmGlu$_5$-GB2	MVLLLILSVLLLKEDVRGSAQSDYKDDDDKSERRVVAHMPGDIIIGALFSVHHQPTVDKVHERKCGAVREQYGIQRVEAMLHTLERINSDPTLLPNITLGCEIRDSCWHSAVALEQSI<br>EFIRDSLISSEEEEGLVRCVDGSSSFRSKKPIVGVIGPGSSSVAIQVQNLLQLFNIPQIAYSATSMDLSDKTLFKYFMRVVPSDAQQARAMVDIVKRYNWTYVSAVHTEGNYGESGM<br>EAFKDMSAKEGICIAHSYKIYSNAGEQSFDKLLKKLRSHLPKARVVACFCEGMTVRGLLMAMRRLGLAGEFLLLGSDGWADRYDVTDGYQREAVGGITIKLQSPDVKWFDDYYLK<br>LRPETNLRNPWFQEFWQHRFQCRLEGFAQENSKYNKTCNSSLTLRTHHVQDSKMGFVINAIYSMAYGLHNMQMSLCPGYAGLCDAMKPIDGRKLLDSLMKTNFTGVSGDMILF<br>DENGDSPGRYEIMNFKEMGKDYFDYINVGSWDNGELKMDDDEVWSKKNNIIRSVCSEPCEKGQIKVIRKGEVSCCWTCTPCKENEYVFDEYTCKACQLGSWPTDDLTGCDLIPV<br>QYLRWGDPEPIAAVVFACLGLLATLFVTVIFIIYRDTPVVKSSSRELCYIILAGICLGYLCTFCLIAKPKQIYCYLQRIGIGLSPAMSYSALVTKTNRIARILAGSKKKICTKKPRFMSACAQ<br>LVIAFILICIQLGIIVALFIMEPPDIMHDYPSIREVYLICNTTNLGVVTPLGYNGLLILSCTFYAFKTRNVPANFNEAKYIAFTMYTTCIIWLAFVPIYFGSNYKIITMCFSVSLSATVALGCMF<br>VPKVYIILAKPERNVRSAFTTSTVVRMHAAATSTSVTSVNQASTSRLEGLQSENHRLRMKITELDKDLEEVTMQLQDTPEKKTN*

Table S1. Primary Structure of Constructs Used in Assays.
Mutations differ between CODA-RET and GABA$_B$-tail assays in the following ways: for mGlu$_1$ glutamate-binding deficient mutants T188A and R78L, respectively, for mGlu$_5$ glutamate-binding deficient mutants, T174A and R68E, respectively, for mGlu$_1$ Gα_q-coupling deficient mutants, F781D and F781S, respectively and for mGlu$_5$ Gα_q-coupling deficient mutants, F767D and F767S, respectively.

Fig Ref	Construct	EC50 µM (±SEM)	Emax AU (±SEM)	n	Fig Ref	Construct	EC50 µM (±SEM)	Emax AU (±SEM)	n
1c	mGlu$_{1/1}$ WT CR	13.91 (13.34-14.48)	56.99 (55.63-58.35)	3	1c	mGlu$_{1/5}$ WT CR	8.42 (6.82-10.02)	56.51 (53.20-59.82)	3
1c	mGlu$_{5/5}$ WT CR	8.81 (7.21-10.41)	50.10 (48.71-51.49)	3	1g	mGlu$_{1/1}$ WT GT	12.9 (12.3-13.5)	73.6 (70.8-76.4)	4
1g	mGlu$_{1/5}$ WT GT	1.51 (1.35-1.70)	96.8 (90.8-102.8)	3	1g	mGlu$_{5/5}$ WT GT	0.96 (0.91-1.00)	83.1 (78.0-88.2)	4
2a	mGlu$_{1/1}$ WT	10.94 (8.33-13.55)	60.74 (57.29-64.19)	3	2a	mGlu$_{1/1}$ Trans	N/A	4.76 (1.77-7.74)	3
2b	mGlu$_{5/5}$ WT	11.24 (9.12-13.36)	53.21 (49.44-56.98)	4	2b	mGlu$_{5/5}$ Trans	3198 (3009-3387)	24.29 (22.93-25.66)	4
2c	mGlu$_{1/5}$ WT	6.53 (5.48-7.58)	53.97 (50.41-57.53)	3	2c	Trans 5=>1	3256 (2431-4081)	20.79 (17.97-23.60)	3
2c	Trans 1=>5	5128 (2943-7313)	18.33 (15.61-21.05)	3	3a	mGlu1/1 WT	13.90 (9.86-17.94)	56.99 (55.63-58.34)	3
3a	mGlu$_{1/1}$ Cis	N/A	6.97 (5.65-8.30)	3	3b	mGlu$_{5/5}$ WT	8.80 (7.19-10.40)	50.11 (48.72-51.50)	3
3b	mGlu$_{5/5}$ Cis	3181 (1574-4787)	27.56 (26.86-28.26)	3	3c	mGlu$_{1/5}$ WT	10.52 (8.34-12.71)	57.03 (54.03-60.06)	10
3c	Cis 1	1094 (979-1208)	38.96 (36.64-41.28)	10	3c	Cis 5	124.3 (77.8-170.9)	10.84 (10.14-11.54)	9
4a	mGlu$_{1/1}$ WT	26.39 (12.87-39.91)	63.15 (56.07-70.23)	7	4a	1 Gα_q only	32.26 (21.06-43.46)	45.15 (38.57-51.72)	7
4b	mGlu$_{5/5}$ WT	15.35 (10.20-20.50)	49.91 (44.52-55.30)	7	4b	5 Gα_q only	16.75 (10.71-22.79)	42.93 (38.64-47.22)	7
4c	mGlu$_{1/5}$ WT CR	8.95 (5.43-12.47)	56.64 (47.72-65.56)	12	4c	Gα_q 1 only CR	10.42 (6.81-14.03)	57.35 (48.96-65.74)	12
4c	Gα_q 5 only CR	19.13 (12.42-25.84)	32.69 (26.42-38.95)	11	4e	mGlu$_{1/5}$ WT GT	1.29 (1.15-1.45)	99.3 (86.9-111.7)	3
4e	Gα_q 1 only GT	2.14 (1.91-2.40)	29.4 (21.3-37.5)	3	4e	Gα_q 5 only GT	2.29 (1.91-2.75)	73.6 (66.4-80.8)	3
5a	mGlu$_{1/1}$ WT	32.14(19.76-44.52)	69.7 (63.44-75.96)	3	5a	mGlu$_5^{1ECD}$/mGlu$_5^{WT}$	15.54 (13.40-17.68)	46.89 (44.53-49.25)	3
5a	mGlu$_5^{1ECD}$ Gα_q only	18.32 (10.44-26.21)	36.68 (33.27-40.09)	3	5a	5 WT Gα_q Only	15.65(10.14-21.16)	34.20 (31.16-37.24)	3
5b	mGlu$_{1/1}$ WT	32.60 (20.74-44.46)	77.83(74.18-81.48)	3	5b	mGlu$_1^{5ECD}$/mGlu$_1^{WT}$	25.49 (17.03-33.95)	73.22(63.36-83.09)	3
5b	mGlu$_1^{5ECD}$ Gα_q only	26.23 (11.76-40.70)	56.13 (46.36-65.90)	3	5b	1 WT Gα_q Only	31.88 (14.52-49.24)	54.57 (49.81-59.34)	3
5c	mGlu$_{1/1}$ WT	30.2 (22.58-37.82)	73.35 (65.23-81.47)	3	5c	mGlu$_5^{1TM}$/mGlu$_5^{WT}$	19.47 (12.48-26.46)	64.89 (52.25-77.53)	3
5c	mGlu$_5^{1TM}$ Gα_q only	21.37 (8.94-33.80)	58.61 (50.21-67.02)	3	5c	5 WT Gα_q Only	27.84(24.81-30.87)	39.63 (29.91-49.35)	3
5d	mGlu$_{1/1}$ WT	34.00 (12.88-55.12)	70.99 (62.76-79.22)	3	5d	mGlu$_1^{5TM}$/mGlu$_1^{WT}$	28.51 (2.78-54.24)	70.87 (62.92-78.82)	3
5d	mGlu$_1^{5TM}$ Gα_q only	43.20 (11.71-74.69)	38.57 (34.86-42.28)	3	5d	1 WT Gα_q Only	19.02 (2.95-35.09)	58.25 (50.02-66.48)	3
6a	mGlu$_{1/1}$ WT +4578	17.79 (7.90-27.68)	80.72 (75.22-86.22)	3	6a	mGlu$_{1/1}$ WT+FITM	ND	8.13 (6.74-9.52)	3
6b	mGlu$_{5/5}$ WT+4578	12.65 (10.17-15.13)	47.26 (45.06-49.46)	3	6b	mGlu$_{5/5}$ WT+FITM	12.65 (10.17-15.13)	47.26 (45.06-49.46)	3
6c	mGlu$_{1/5}$ WT +4578	7.81 (2.26-13.35)	66.56 (57.77-75.35)	9	6c	mGlu$_{1/5}$ WT +FITM	23.84 (14.12-33.56)	36.82 (30.5-43.13)	6
6d	Gα_q 1 only+4578	7.83 (3.08-12.58)	70.52 (62.12-78.92)	9	6d	Gα_q 1 only+FITM	ND	2.72 (-0.01-5.45)	6
6e	Gα_q 5 only+4578	14.12 (5.91-22.33)	23.9 (20.13-27.67)	9	6e	Gα_q 5 only+FITM	31.75 (22.85-40.64)	41.72 (36.29-47.15)	6
6f	mGlu$_{1/5}$ WT+MTEP	13.72 (7.81-19.63)	50.56 (41.41-59.71)	5	6g	Gα_q 1 only+MTEP	12.48 (8.96-16.00)	49.1 (43.20-55.20)	5
6h	Gα_q 5 only+MTEP	32.41 (13.86-50.96)	8.60 (5.04-12.17)	4	7a	mGlu$_{1/1}$ WT	37.09 (9.14-65.04)	69.80 (60.98-78.63)	5
7a	mGlu$_{1/1}$ WT + 4578	11.82 (5.36-18.28)	89.31(81.19-97.44)	4	7b	mGlu$_1^{Y672V}$/mGlu$_1^{Y672V}$	25.6 (4.25-46.95)	62.07 (54.26-69.88)	5
7b	mGlu$_1^{Y672V}$/mGlu$_1^{Y672V}$ + 4578	15.57 (4.77-26.37)	63.55 (54.88-72.22)	4	7c	mGlu$_1^{Y672V,F781D}$/mGlu$_1$	19.29 (7.51-31.07)	42.51 (36.87-48.14)	4
7c	mGlu$_1^{Y672V,F781D}$/mGlu$_1$ +4578	15.99 (4.58-27.40)	68.39 (62.69-74.10)	4	7d	mGlu$_1^{Y672V}$/mGlu$_1^{F781D}$	29.86 (11.72-48.00)	41.88 (32.08-51.68)	4
7d	mGlu$_1^{Y672V}$/mGlu$_1^{F781D}$ + 4578	27.62 (10.84-44.40)	42.6 (33.45-51.75)	4	7e	mGlu$_{1/1}$ WT	36.05 (23.92-48.18)	66.08 (58.26-73.90)	4
7e	mGlu$_{1/1}$ WT + FITM	41.51 (26.97-56.06)	21.58 (19.51-23.65)	4	7f	mGlu$_1^{G665F}$/mGlu$_1^{G665F}$	47.23 (35.10-59.36)	37.80 (34.61-40.99)	4
7f	mGlu$_1^{G665F}$/mGlu$_1^{G665F}$+ FITM	47.32 (35.58-59.06)	36.86 (33.89-39.83)	4	7g	mGlu$_1^{G665F,F781D}$/mGlu$_1$	15.99 (4.58-27.40)	68.39 (62.69-74.10)	4
7g	mGlu$_1^{G665F,F781D}$/mGlu$_1$ +FITM	36.84 (22.63-51.05)	16.83 (11.26-22.40)	4	7h	mGlu$_1^{G665F}$/mGlu$_1^{F781D}$	41.96 (36.03-47.89)	22.25 (14.44-30.06)	4
7h	mGlu$_1^{G665F}$/mGlu$_1^{F781D}$ +FITM	47.36 (33.51-61.21)	23.26 (15.52-31.00)	4	Ext 1a	mGlu$_1$ WT	2.37 (1.95-2.79)	1.37 x 10^{-6} (0.84-1.91)	4
Ext 1a	mGlu$_1$-HiBiT	2.69 (2.37-3.01)	1.35 x 10^{-6} (0.90-1.80)	4	Ext 1a	mGlu$_1$-LgBiT	2.54 (2.00-3.08)	1.31 (1.04-1.57)	4
Ext 1c	mGlu$_5$ WT	1.66 (0.60-2.72)	1.13 x 10^{-6} (0.46-1.81)	3	Ext 1c	mGlu$_5$-HiBiT	2.97 (0.27-5.68)	1.09 x 10^{-6} (0.36-1.82)	3
Ext 1c	mGlu$_5$-LgBiT	2.24 (1.44-3.04)	0.92 x 10^{-6} (0.49-1.35)	3	Ext 1e	mGlu$_1$ WT	6.62 (-0.25 – 13.49)	3.36 x 10^{-5} (1.47-5.24)	4
Ext 1e	mGlu$_1^{T188A}$	ND	-2.1 x 10^{-4} (-4.41-0.25)	4	Ext 1e	mGlu$_1^{F781D}$	ND	-0.37 x 10^{-4} (-4.3-3.6)	4
Ext 1e	mGlu$_1^{T188A,F781D}$	ND	-0.18 x 10^{-4} (-3.7-0.05)	4	Ext 1e	mGlu$_1$-HiBiT	7.42 (-0.21-15.05)	3.66 x 10^{-5} (0.94-6.37)	3

Fig Ref	Construct	EC50 µM (±SEM)	Emax AU (±SEM)	n	Fig Ref	Construct	EC50 µM (±SEM)	Emax AU (±SEM)	n
Ext 1e	mGlu$_1^{T188A}$-HiBiT	ND	-3.2 x 10^{-4} (-4.7-(-)1.6)	3	Ext 1e	mGlu$_1^{F781D}$-HiBiT	ND	-3.3 x 10^{-4} (-6.3- (-) 0.2)	3
Ext 1e	mGlu$_1^{T188A,F781D}$-HiBiT	ND	-1.0 x 10^{-4} (-1.9-(-)0.2)	3	Ext 1e	mGlu$_1$-LgBiT	8.92 (-0.74-18.6)	5.02 x 10^{6} (2.07-7.97)	4
Ext 1e	mGlu$_1^{T188A}$-LgBiT	ND	-1.5 x 10^{-4} (-3.2 - 0.01)	4	Ext 1e	mGlu$_1^{F781D}$-LgBiT	ND	-0.9 x 10^{-4} (-3.4 – 1.5)	4
Ext 1e	mGlu$_1^{T188A,F781D}$-LgBiT	ND	-2.1 x 10^{-4} (-3.2- (-)1.0)	4	Ext 1f	mGlu$_5$ WT	1.48 (-0.31 – 3.27)	1.95 x 10^{-5} (-0.3-4.24)	4
Ext 1f	mGlu$_5^{T174A}$	ND	2.1 x 10^{-4} (0.4-3.7)	3	Ext 1f	mGlu$_5^{F767D}$	ND	-1.2 x 10^{-4} (-3.2-0.7)	3
Ext 1f	mGlu$_5^{T174A,F767D}$	ND	-1.2 x 10^{-4} (-2.3-(-)0.06)	4	Ext 1f	mGlu$_5$-HiBiT	7.53 (-0.91-16.0)	3.00 x 10^{-5} (2.42-3.59)	3
Ext 1f	mGlu$_5^{T174A}$-HiBiT	ND	2.8 x 10^{-4} (0.3-5.2)	3	Ext 1f	mGlu5$_{F781D}$-HiBiT	ND	-3.4 x 10^{-4} (-8.1-1.4)	3
Ext 1f	mGlu$_5^{T174A,F767D}$-HiBiT	ND	-2.4 x 10^{-4} (-4.3-(-)0.5)	3	Ext 1f	mGlu$_5$-LgBiT	3.69 (-0.21-7.59)	4.33 x 10^{6} (2.43-6.23)	3
Ext 1f	mGlu$_5^{T174A}$-LgBiT	ND	-1.4 x 10^{-4} (-4.6 - 1.9)	3	Ext 1f	mGlu$_5^{F767D}$-LgBiT	ND	-4.1 x 10^{-4} (-7.8 – (-)0.3)	3
Ext 1f	mGlu$_5^{T174A,F767D}$-LgBiT	ND	-0.3 x 10^{-4} (-1.2- 0.6)	3	Ext 2a	mGlu$_{1/5}$ WT	10.3 (6.8-13.8)	52.6 (45.1-60.1)	4
Ext 2a	mGlu$_{1/5}$ WT + mGlu$_1$ WT	13.4 (9.5-17.3)	6.59 (4.63-8.55)	4	Ext 2a	mGlu$_{1/5}$ WT + mGlu$_5$ WT	34.3 (18.2-50.4)	4.51 (3.42-5.61)	4
Ext 3a	mGlu$_1$-GB1/GB2 WT	12.9 (12.3-13.5)	73.6 (70.8-76.4)	4	Ext 3a	mGlu$_1^{R78L}$/mGlu$_1^{F781S}$-GB1/GB2	ND	28.0 (24.1-31.9)	3
Ext 3b	mGlu$_5$-GB1/GB2 WT	0.96 (0.91-1.00)	83.1 (78.0-88.2)	4	Ext 3b	mGlu$_5^{R68E}$/mGlu$_5^{F767S}$-GB1/GB2	141 (123-162)	89.9 (83.3-96.5)	3
Ext 3c	mGlu$_{1/5}$-GB1/GB2 WT	1.58 (1.55-1.62)	94.5 (89.6-99.6)	3	Ext 3c	mGlu1$_{R78L}$/5$_{F767S}$-GB1/GB2	251 (234-269)	134.9 (119.8-150.0)	3
Ext 3c	mGlu$_1^{F781S}$ /mGlu$_5^{R68E}$-GB1/GB2	417 (380-457)	18.2 (16.6-19.8)	3	Ext 3d	mGlu$_1^{R78L,F781S}$/mGlu$_1^{WT}$-GB1/GB2	ND	28.9 (24.9=32.9)	3
Ext 3e	mGlu$_1^{R78L,F781}$/mGlu$_5$-GB1/GB2	ND	ND	3	Ext 3e	mGlu$_1^{WT}$/mGlu$_5^{R68E\ ,F767S}$-GB1/GB2	426 (371-490)	62.1 (52.5-71.7)	3
Ext 4a	mGlu$_{1/5}$-GB1/GB2 + DMSO	1.28 (1.15-1.45)	99.3 (86.9-111.7)	3	Ext 4a	mGlu$_{1/5}$-GB1/GB2 + FITM	2.29 (2.09-2.51)	59.2 (49.6-68.8)	3
Ext 4a	mGlu$_{1/5}$-GB1/GB2 + 4578	0.78 (0.76-0.79)	112.7 (100.5-124.9)	3	Ext 4b	mGlu$_1$/mGlu$_5^{F767S}$-GB1/GB2 + DMSO	2.29 (1.91-2.75)	73.6 (66.4-80.8)	3
Ext 4b	mGlu$_1$/mGlu$_5^{F767S}$-GB1/GB2 + FITM	ND	0.7 (-0.1-0.8)	3	Ext 4b	mGlu$_1$/mGlu$_5^{F767S}$-GB1/GB2 + 4578	0.89 (0.79-1.00)	108.9 (102.6- 115.2)	3
Ext 4c	mGlu$_1^{F781S}$/mGlu$_5$-GB1/GB2+ DMSO	2.14 (1.91-2.40)	29.4 (21.3-37.5)	3	Ext 4c	mGlu$_1^{F781S}$/mGlu$_5$-GB1/GB2 + FITM	1.74 (1.70-1.78)	105.5 (99.8-111.2)	3
Ext 4c	mGlu$_1^{F781S}$/mGlu$_5$-GB1/GB2 + 4578	1.41 (1.12-1.78)	14.0 (6.6-21.4)	3	Ext 5a	mGlu$_{1/5}$* WT	5.84 (5.33-6.35)	48.51 (47.02-50.00)	3
Ext 5b	mGlu$_{1/5}$* CisTrans	10.3 (5.9-14.6)	57.95 (49.38-66.52)	9	Ext 5b	Gα_q 1* only	11.7 (6.8-16.6)	56.73 (49.43-64.03)	9
Ext 5b	Gα_q 5* only	18.1 (11.5-24.7)	33.21 (27.31-39.11)	9	Ext 5c	Gα_q 1* only+4578	6.55 (4.37-8.74)	71.15 (62.47-79.83)	6
Ext 5c	Gα_q 1* only+FITM	NA (N/A)	0.60 (-2.67-3.87)	4	Ext 5d	Gα_q 5* only+4578	10.48 (6.77-14.19)	23.71 (16.06-31.36)	6
Ext 5d	Gα_q 5* only+FITM	27.83 (22.76-32.90)	42.44 (33.98-50.90)	4	Ext 5e	mGlu$_{1/5}$* CisTrans+MTEP	12.64 (7.41-17.87)	45.2 (33.31-57.09)	5
Ext 5f	Gα_q 1* only+MTEP	16.03 (9.87-22.19)	52.12 (37.02-67.22)	5	Ext 5g	Gα_q 5* only+MTEP	NA (NA)	6.20 (2.70-9.70)	5

Table S2. EC_{50}'s and E_{max}'s for All Assays.
In parentheses, ±SEM for EC_{50} and E_{max} and number for repeats (n). CR = CODA-RET assay; GT = $GABA_B$-tail assay.

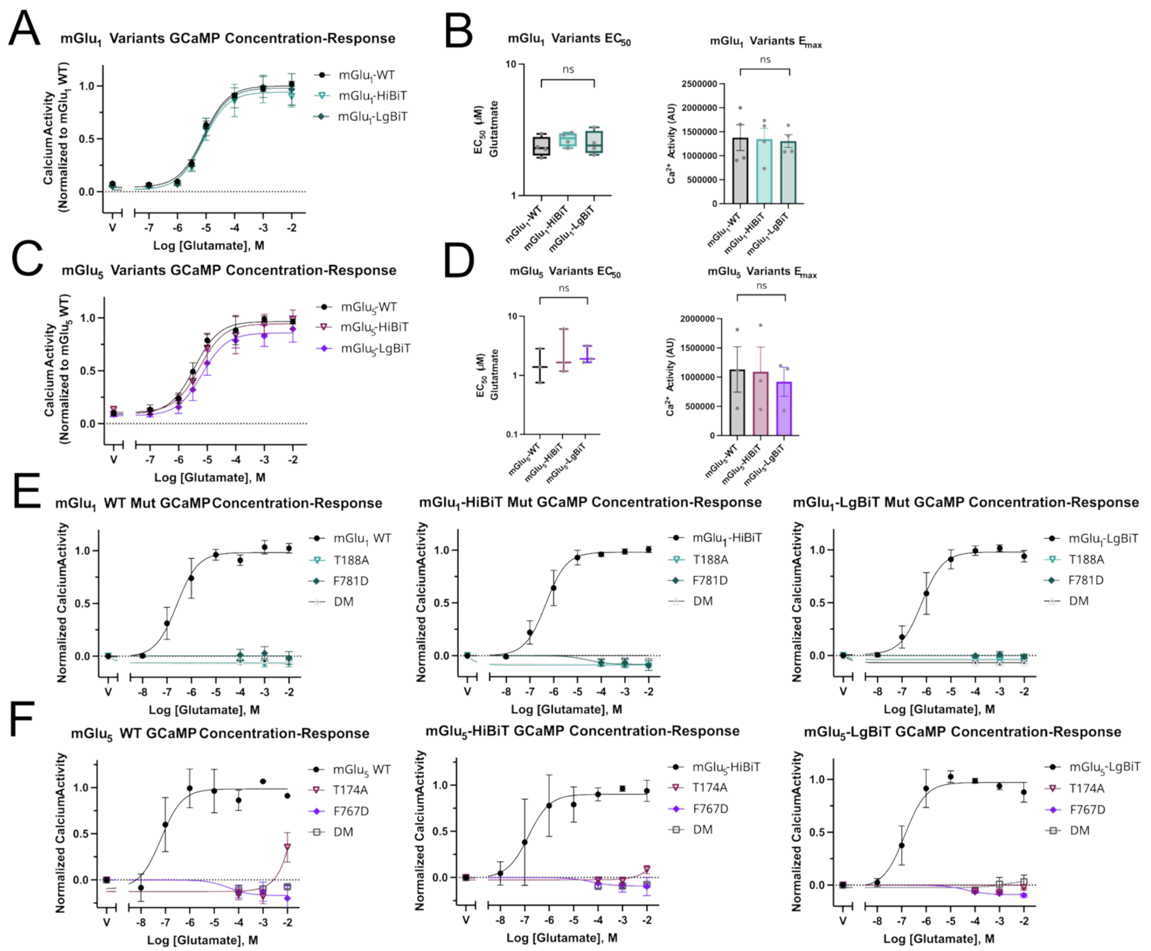

Figure S1. Validation of Variants Using GCaMP.
(A) Left shows a concentration-response curve of $mGlu_1$ WT with vehicle (black), $mGlu_1$-HiBiT (turquoise), and $mGlu_1$-LgBiT (teal).
(B) Left shows corresponding EC_{50}: ns 0.27 = and ns = 0.64. Rights shows corresponding E_{max}: ns = 0.93 and ns= 0.82.
(C) Left shows a concentration-response curve of $mGlu_5$ WT with vehicle (black), $mGlu_5$-HiBiT (magenta), and $mGlu_5$-LgBiT (purple).
(D) Left shows corresponding EC_{50}: ns 0.48 = and ns = 0.49. Rights shows corresponding E_{max}: ns = 0.95 and ns= 0.67.
(E) Concentration-response curves for $mGlu_1$ WT, $mGlu_1$-HiBiT, and $mGlu_1$-LgBiT (left to right-black) compared with orthosteric binding mutant T188A (turquoise), $G\alpha_q$-binding mutant F781D (teal), and double mutant (DM) T188A/F781D (grey).
(F) Concentration-response curves for $mGlu_5$ WT, $mGlu_5$-HiBiT, and $mGlu_5$-LgBiT (left to right-black) compared with orthosteric binding mutant T174A (magenta), $G\alpha_q$-binding mutant F767D (purple), and double mutant (DM) T174A/F767D (grey). Symbols represent the mean drug-induced GCAMP fluorescence and error bars represent ± SEM. The exact number of 'n' independent experiments and technical replicates are reported in Table S2.

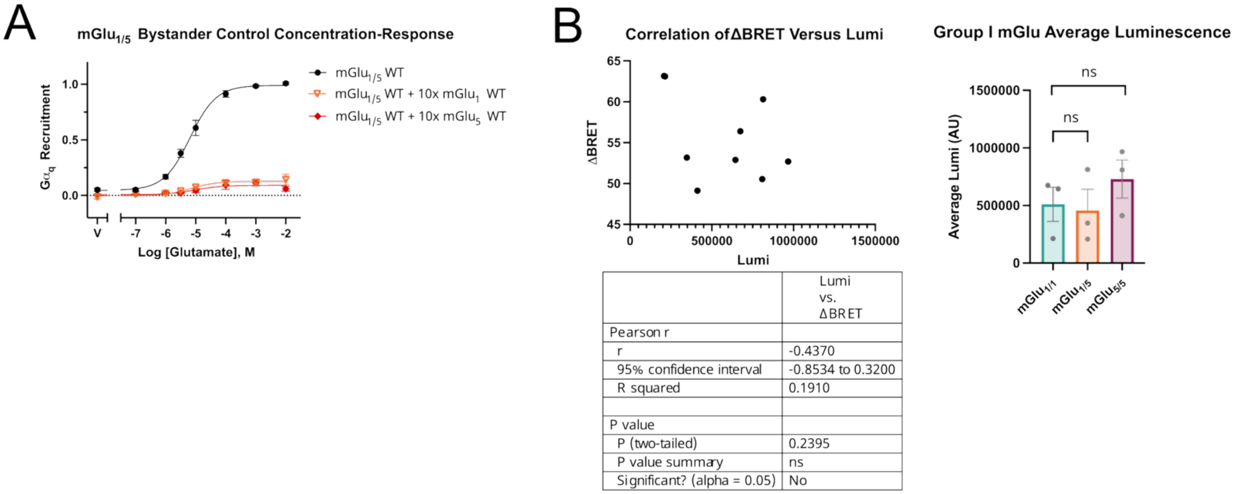

	Lumi vs. ΔBRET
Pearson r	
r	-0.4370
95% confidence interval	-0.8534 to 0.3200
R squared	0.1910
P value	
P (two-tailed)	0.2395
P value summary	ns
Significant? (alpha = 0.05)	No

Figure S2. CODA-RET Assay Validation.
(A) Concentration-response curve of $mGlu_{1/5}$ WT (50 ng $mGlu_1$-LgBiT and 100 ng $mGlu_5$-HiBiT) (black), with 500 ng of $mGlu_1$ WT (light orange), or with 1000 ng $mGlu_5$ WT (dark orange).
(B) Left shows a correlation plot of ΔBRET versus Luminescence showing an r = -0.4370 and p = 0.24. Rights shows a plot of average luminescence for the data from Figure 1. ns = 0.83 and 0.38.

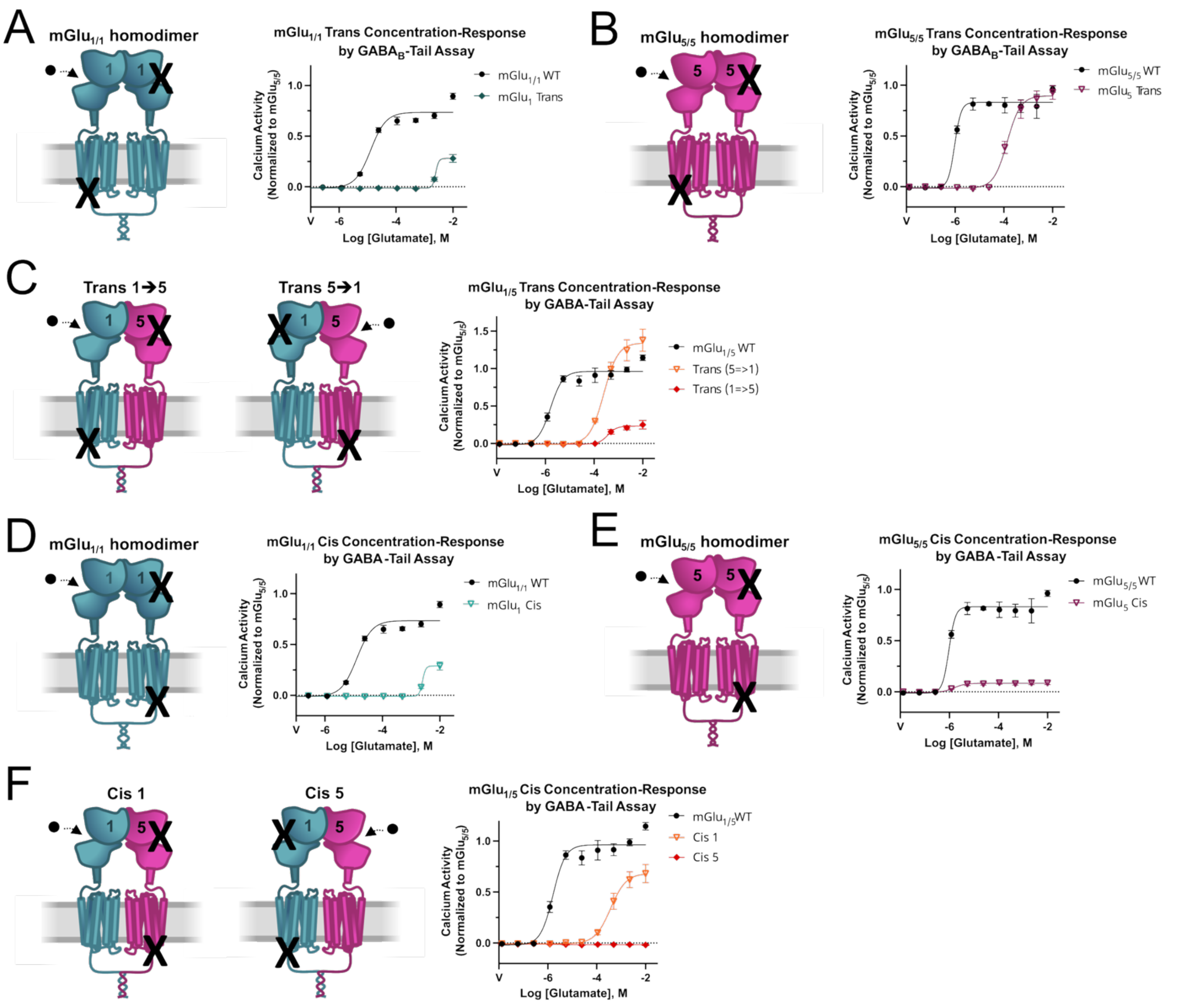

Figure S3. GABA$_B$-tails Assay Confirmation of Trans- and Cis-activation.

(A) Left shows schematic of R78L and F781S mutations on one mGlu$_1$-GB1 protomer paired with mGlu$_1$-GB2 WT (cyan), restricting to transactivation. Right shows a concentration-response curve of mGlu$_{1/1}$-GB1/GB2 (black) versus mGlu$_{1/1}$ in trans (cyan) by GABA$_B$-tail assay.

(B) Left shows schematic of R68E and F767S mutations on one mGlu$_5$-GB1 protomer paired with mGlu$_5$-GB2 WT (cyan), restricting to transactivation. Right shows a concentration-response curve of mGlu$_{5/5}$-GB1/GB2 WT (black) versus mGlu$_{1/1}$-GB1/GB2 in trans (cyan) by GABA$_B$-tail assay.

(C) Left shows schematic of R78L mutation on mGlu$_1$-GB1 protomer (cyan) and F767S mutation on mGlu$_5$-GB2 protomer (magenta), restricting to transactivation from mGlu$_5$ to mGlu$_1$. Middle shows schematic of F781S mutation on mGlu$_1$-GB1 protomer (cyan) and R68E mutation on mGlu$_5$-GB2 protomer (magenta), restricting to transactivation from mGlu$_1$ to mGlu$_5$. Right shows a concentration-response curve of mGlu$_{1/5}$-GB1/GB2 (black) versus mGlu$_{1/5}$-GB1/GB2 in trans from 5 to 1 (light orange) versus mGlu$_{1/5}$ in trans from 1 to 5 (dark orange) by GABA$_B$-tail assay.

(D) Left shows schematic of R78L and F781S mutations on one mGlu$_1$-GB1 protomer paired with mGlu$_1$-GB2 WT (cyan), restricting to cis activation. Right shows a concentration-response curve of mGlu$_{1/1}$-GB1/GB2 WT (black) versus mGlu$_{1/1}$ -GB1/GB2 in cis (cyan) by GABA$_B$-tail assay.

(E) Left shows schematic of R68E and F767S mutations on one $mGlu_5$-GB1 protomer paired with $mGlu_5$-GB2 (magenta), restricting to cis activation. Right shows a concentration-response curve of $mGlu_{5/5}$-GB1/GB2 (black) versus mGlu5-GB1/5-GB2 in cis (magenta) by $GABA_B$-tail assay.
(F) Left shows schematic of R68E and F767S mutations on $mGlu_5$ protomer (magenta) paired with $mGlu_1$ WT, restricting to cis activation through $mGlu_1$. Middle shows R78L and F781S mutations on mGlu-GB1 protomer (cyan) paired with $mGlu_5$-GB2 WT (magenta), restricting to cis activation through $mGlu_5$. Right shows a concentration-response curve of $mGlu_{1/5}$-GB1/GB2 (black) versus $mGlu_{1/5}$-GB1/GB2 in cis through $mGlu_1$ (light orange) versus $mGlu_{1/5}$-GB1/GB2 in cis through mGlu5 (dark orange) by $GABA_B$-tail assay. The exact number of 'n' independent experiments and technical replicates are reported in Table S2.

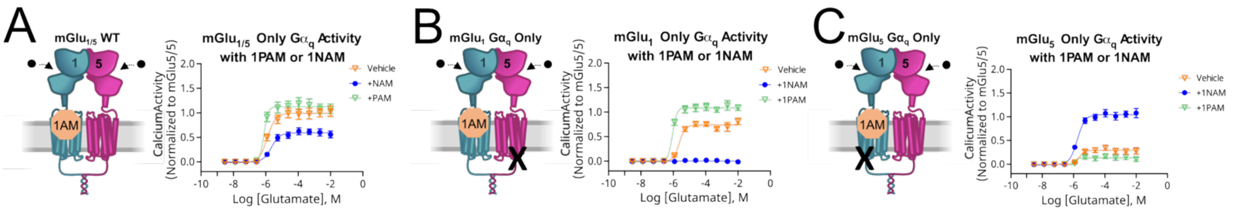

Figure S4. $GABA_B$-tail Assay Confirmation of Inversion of $mGlu_1$ PAM and NAM Signaling.
(A) Left shows a schematic of $mGlu_{1/5}$-GB1/GB2 with a 1PAM bound to the allosteric binding pocket of $mGlu_1$. Right shows a concentration-response curve of $mGlu_{1/5}$-GB1/GB2 with vehicle (orange), with PAM (100 nM VU6024578-green), or with NAM (100 nM FITM-blue).
(B) Left shows a schematic of $mGlu_{1/5}$-GB1/GB2 with a $G\alpha_q$-binding point mutation (F767S) in $mGlu_5$ with a 1PAM bound to the allosteric binding pocket of $mGlu_1$. Right shows a concentration-response curve of $mGlu_{1/5}$-GB1/GB2 with vehicle (grey), $mGlu_1$/$mGlu5^{F767S}$ with vehicle (orange), $mGlu_1$/$mGlu5^{F767S}$ with PAM (100 nM VU6024578-green), or $mGlu_1$/$mGlu5^{F767S}$ with NAM (100 nM FITM-blue).
(C) Left shows a schematic of $mGlu_{1/5}$-GB1/GB2 with a $G\alpha_q$-binding point mutation (F781S) in $mGlu_1$ with a 1PAM bound to the allosteric binding pocket of $mGlu_1$. Middle shows a concentration-response curve of $mGlu_{1/5}$-GB1/GB2 (grey) with vehicle, $mGlu_1^{F781S}$/$mGlu_5$ with vehicle (orange), $mGlu_1^{F781S}$/$mGlu_5$ with PAM (100 nM VU6024578-green), or $mGlu_1^{F781S}$/$mGlu_5$ with NAM (100 nM FITM-blue). The exact number of 'n' independent experiments and technical replicates are reported in Table S2.

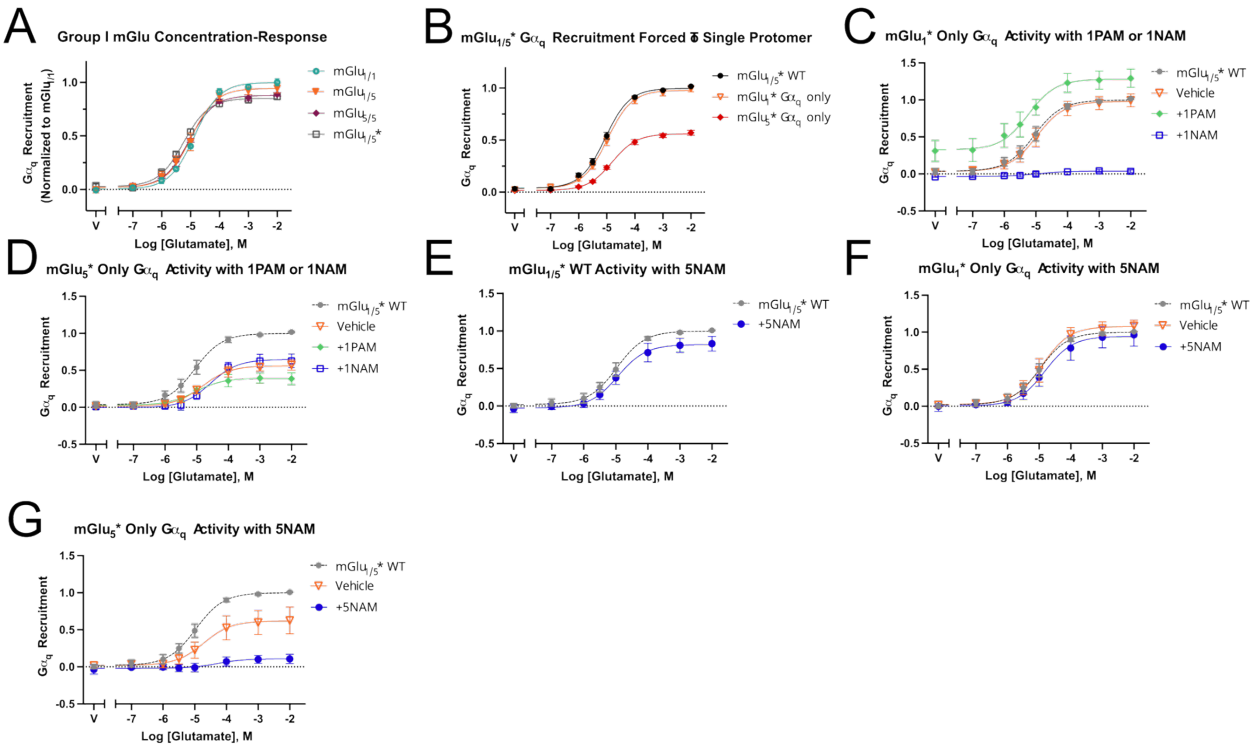

Figure S5. Polarity Repeats of Key Experiments.

mGlu$_1$-LgBiT and mGlu$_5$-HiBiT were used for all of the heterodimer experiments in the primary text due to very slight deficits in mGlu$_5$-LgBiT activity. Here are repeats of the key experiments using the heterodimer with mGlu$_1$-HiBiT/mGlu$_5$-LgBiT (mGlu$_{1/5}$*).

(A) Concentration-response curve from Figure 1 with mGlu$_{1/5}$* WT overlayed (black box).

(B) Concentration-response curve of mGlu$_{1/5}$* WT (black) versus mGlu$_1$/mGlu$_5^{F767D}$* (light orange) versus mGlu$_1^{F781D}$/mGlu$_5$* (dark orange) corresponding to Figure 4 in the main text.

(C) Concentration-response curve of mGlu$_{1/5}$* WT (grey) versus mGlu$_1$/mGlu$_5^{F767D}$* (orange) with PAM (100nM VU6024578-green) or NAM (100nM FITM-blue) corresponding to Figure 6.

(D) Concentration-response curve of mGlu$_{1/5}$* WT (grey) versus mGlu$_1^{F781D}$/mGlu$_5$* (orange) with PAM (100nM VU6024578-green) or NAM (100nM FITM-blue) corresponding to Figure 6.

(E) Concentration-response curve of mGlu$_{1/5}$* WT with vehicle (grey), with NAM (1 uM MTEP-blue).

(F) Concentration-response curve of mGlu$_{1/5}$* WT with vehicle (grey), mGlu$_1$/mGlu$_5^{F767D}$* with vehicle (orange), and mGlu$_1$/mGlu$_5^{F767D}$* with NAM (1uM MTEP-blue).

(G) Concentration-response curve of mGlu$_{1/5}$* WT with vehicle (grey), mGlu$_1^{F781D}$/mGlu$_5$* with vehicle (orange), and mGlu$_1^{F781D}$/mGlu$_5$* with NAM (1 uM MTEP-blue). Symbols represent the mean drug-induced BRET response and error bars represent ± SEM. The exact number of ‘n’ independent experiments and technical replicates are reported in Table S2.
